# The injury mechanisms and injury pyramids among children and adolescents in Zhuhai City, China

**DOI:** 10.1186/s12889-021-10425-4

**Published:** 2021-03-04

**Authors:** Xiling Yin, Wencan Dai, Yukai Du, Deyun Li

**Affiliations:** 1 Department of Public Health and Health Research, Center for Disease Control and Prevention of Zhuhai City, Zhuhai, China; 2 Department of Maternal and Child Health, School of Public Health, Tongji Medical College, Huazhong University of Science and Technology, Wuhan, China

**Keywords:** Injury, Children, Adolescents, Injury mechanisms, Injury pyramid, China

## Abstract

**Background:**

The unclear mechanisms and severity of injuries in the injury pyramids for Chinese children and adolescents prevent the prioritization of interventions. This study aimed to describe the injury mechanisms and injury pyramids in this population to provide a priority for injury prevention strategies.

**Methods:**

Death, hospitalization, and outpatient/emergency department visit data from patients aged 0 ~ 17 years with injuries were obtained from January 1, 2013, to December 31, 2017, in Zhuhai City, China. The injury mechanism ratios were calculated, and the injury pyramid ratios were drawn in proportion using injury mortality and the incidence of both injury hospitalizations and outpatient/emergency department injury visits.

**Results:**

The top three mechanisms for injuries in children and adolescents treated in outpatient/emergency departments were falls (52.02%), animal bites (14.57%), and blunt injuries (10.60%). The top three mechanisms for injury hospitalizations were falls (37.33%), road traffic injuries (17.87%), and fire/burns (14.29%), while the top three mechanisms for injury deaths were drowning (32.91%), road traffic injuries (20.25%) and falls (13.92%). The incidence rate of outpatient/emergency department injury visits for children and adolescents was 11,210.87/100,000; the incidence rate of injury hospitalization was 627.09/100,000, and the injury death rate was 10.70/100,000. For each injury death, there were 59 injury hospitalizations and 1048 outpatient/emergency injury visits.

**Conclusions:**

The injury mechanisms were different for injury-related outpatient/emergency department visits, hospitalizations, and deaths among children and adolescents. The injury mechanisms by sex at different stages of child development, and interventions should be formulated based on this finding. The ratios of the injury pyramids varied by age, sex, region, and injury mechanisms; minor nonfatal injuries were more common in children and adolescents. The differences in the severity and extent of the injuries suggested that injury interventions in children and adolescents still have a long way to go.

## Background

Children and adolescents are high-risk groups and key populations for injury prevention [1]. In recent years, the global injury mortality rate of children has declined overall, but the phenomenon of inequality in injury mortality between high-income countries (HICs) and lower-middle- and upper-middle-income countries (LMICs) has expanded [2]. Road traffic injuries were the leading cause of child deaths worldwide, followed by drowning [3]. According to China’s monitoring data for 2014, the death rate for children and adolescents aged 0–17 was 22.90/100,000. Approximately 65,000 children died from injuries each year. Injury was the leading cause of death for children aged 1–17. Approximately 140,000 children suffer from nonfatal injuries; the main cause of death among Chinese children and adolescents is drowning and road traffic injuries [4]. The injury mortality rate or incidence rate, mechanism of occurrence, and injury patterns of different countries, regions and data sources vary widely [5–7].

Although mortality is often used as a criterion for understanding the severity level of an injury, this approach represents an “iceberg” effect for injury. The severity of an injury may vary depending on the injury mechanisms, demographic characteristics, and prognosis; therefore, it is not possible to fully understand injury status through mortality or a single source of injury statistics [8]. The bottom of the injury pyramid to the top includes the injury cases treated in outpatient/emergency departments, injury hospitalizations, and injury deaths. Depicting the “injury pyramid” can help us understand and grasp the whole picture of the injury. Lee [9] described the injury pyramid ratio for children and adolescents living in Massachusetts in the United States as 1:36:1014. The injury pyramid ratio among South Korean teenagers (13–18 years old) was 1:162:492 [10]. The unintentional injury pyramid ratio in South Korea was 1:54:231 [8]. The injury pyramid ratio of the Missouri and Nebraska population of the United States was 1:10:147 [11]. Based on family surveys, the injury pyramid ratio in Iran was 1:50:646 [12]. The injury pyramid ratios vary among different countries, regions, and populations. In some areas of China, research on the injury pyramids of the whole population has been carried out [13, 14], but to date, there have been no reports on the injury pyramid for Chinese children and adolescents. The unclear mechanisms and severity of the injuries in the injury pyramids for Chinese children and adolescents prevent the prioritization of interventions.

The comprehensive use of injury information that integrates deaths, hospitalizations, and outpatient/emergency department visits can provide a reliable basis for the development of injury prevention strategies. Continuous and systematic collection of injury-related information and standardized comprehensive injury monitoring databases have resulted in the accumulation of authoritative, valuable first-hand data for the analysis of injury spectra and pyramids for Chinese children and adolescents.

## Methods

### Settings

Zhuhai City, one of the monitoring points of the Chinese National Injury Surveillance System (NISS), is one of the core cities in the Greater Bay Area of Guangdong, Hong Kong and Macau, and one of the leading frontier cities in China’s globalization process. The land area of Zhuhai City is 1711.24 km^2^. The average number of resident population in 2013–2017 was 16,315,60, of which males accounted for 53.21%, the urban population accounted for 55.10%, the floating population accounted for approximately 31.30%, and the population aged 0–17 accounted for 18.10%. Zhuhai City has three administrative districts: Xiangzhou District is the city’s main urban area, and Jinwan District and Doumen District are the rural areas in the western region. In recent years, with the increasing saturation of residential, industrial and commercial land in Xiangzhou District, Jinwan District and Doumen District are experiencing rapid population growth and accelerating urbanization.

There are three sentinel monitoring hospitals in Zhuhai City that are included in the NISS for collecting outpatient/emergency department injury cases. Of these three hospitals, 2 of them are distributed in urban areas, and 1 is in a rural area; they are government-sponsored comprehensive hospitals with the ability to treat patients with emergency injuries. The hospitalization data obtained in this study cover 24 hospitals that can provide hospitalization for injuries. There are 15 hospitals in urban areas and 9 hospitals in rural areas. The hospitals’ diagnosis and treatment cover all permanent residents, including both floating and household populations. The death data came from the Chinese national population death information registration management system, which also covers all permanent residents.

### Data collection

The period for obtaining data was from January 1st, 2013, to December 31st, 2017.

#### Outpatient/emergency departments

Injured patients aged 0 ~ 17 years who first visited an outpatient/emergency department in one of the three injury monitoring hospitals in Zhuhai City were selected as subjects. The personal information of the injured patient, the basic situation of the injury event, and the clinical information related to the injuries were derived from the NISS.

#### Hospitalization

The inpatients who were 0 ~ 17 years old, had experienced their first hospitalization because of injuries and were diagnosed with an injury (indicated by an external cause code of V01-Y98) according to The International Statistical Classification of Diseases and Related Health Problems 10th Revision (ICD-10) were selected as subjects (Table 1). The data came from the Medical Information Management Platform of Zhuhai City, which collected information such as personal information on inpatients and clinical features of injuries. There were 24 hospitals covering all residents in the city with inpatient treatment wards.

**Table 1:** The mechanisms of injuries and ICD-10 code

Mechanisms of injuries	ICD-10 code
Road traffic injuries	V01-V89
Falls	W00-W19
Bruise/crush/glass or knife cut/machine accident	W20-W31
Foreign matter entering the eye or other cavity/cutting and puncture device injuries	W41-W49
Boxing and punching injuries in sports	W50- W52
Animal bites	W53-W59
Drowning	W65-W74
Asphyxiation	W75-W84
Fires and burns	X00-X19
Poisoning	X20-X29,X40-X49
Overwork/travel and poverty	X50- X57
Self-harm/suicide	X60-X84
Harm by others	X85-Y09
Drug reaction/medical accident/surgery and medical complications	Y40-Y84
Others	W32-W40, W60, W64, W85-W99, V90-V99, X30-X39, X58-X59, Y10-Y34, Y35-Y36, Y85-Y89,Y90-Y98

#### Death

The death data came from the Chinese national population death information registration management system. According to their current address, all the children and adolescents aged 0–17 who died from injuries (root causes of death codes V01-Y98) in the resident population were identified. The data collected by this registry system included the personal information of the deceased, the place of death, and the basis causes of disease diagnosis.

#### Population data

The population data of residents 0 to 17 years old from 2013 to 2017 were derived from the basic information system in the Chinese disease prevention information control system. In the resident population data, there was a specific value for every 1 year of age between 0 and 9 years, but summations were provided for the children/adolescents aged 10 to 14 and 15 to 19 years old. We divided the population of subjects aged 10 to 14 years old and 15 to 19 years old evenly and added them to the specific populations of other age groups to calculate the population numbers for the subjects aged 6 to 11 years, 12 to 14 years and 15 to 17 years (Table 2).

**Table 2:** Number of residents aged 0–17 in Zhuhai City, 2013 ~ 2017

Years	Gender		Region		Age group (years old)					Total
	Male	Female	Urban	Rural	0 ~ 2	3 ~ 5	6 ~ 11	12 ~ 14	15 ~ 17	
2013	156,000	132,045	165,540	122,505	44,367	36,315	84,196	44,647	78,520	288,045
2014	155,127	133,815	166,034	122,908	44,505	36,415	84,444	44,774	78,804	288,942
2015	162,682	128,763	167,556	123,889	44,894	36,783	85,243	45,223	79,302	291,445
2016	159,388	136,586	170,113	125,861	45,170	37,394	86,700	45,976	80,734	295,974
2017	176,769	135,017	158,304	153,482	49,305	53,957	93,816	49,727	64,981	311,786

### Statistics of the injury pyramid ratio

**Fig. 1:**
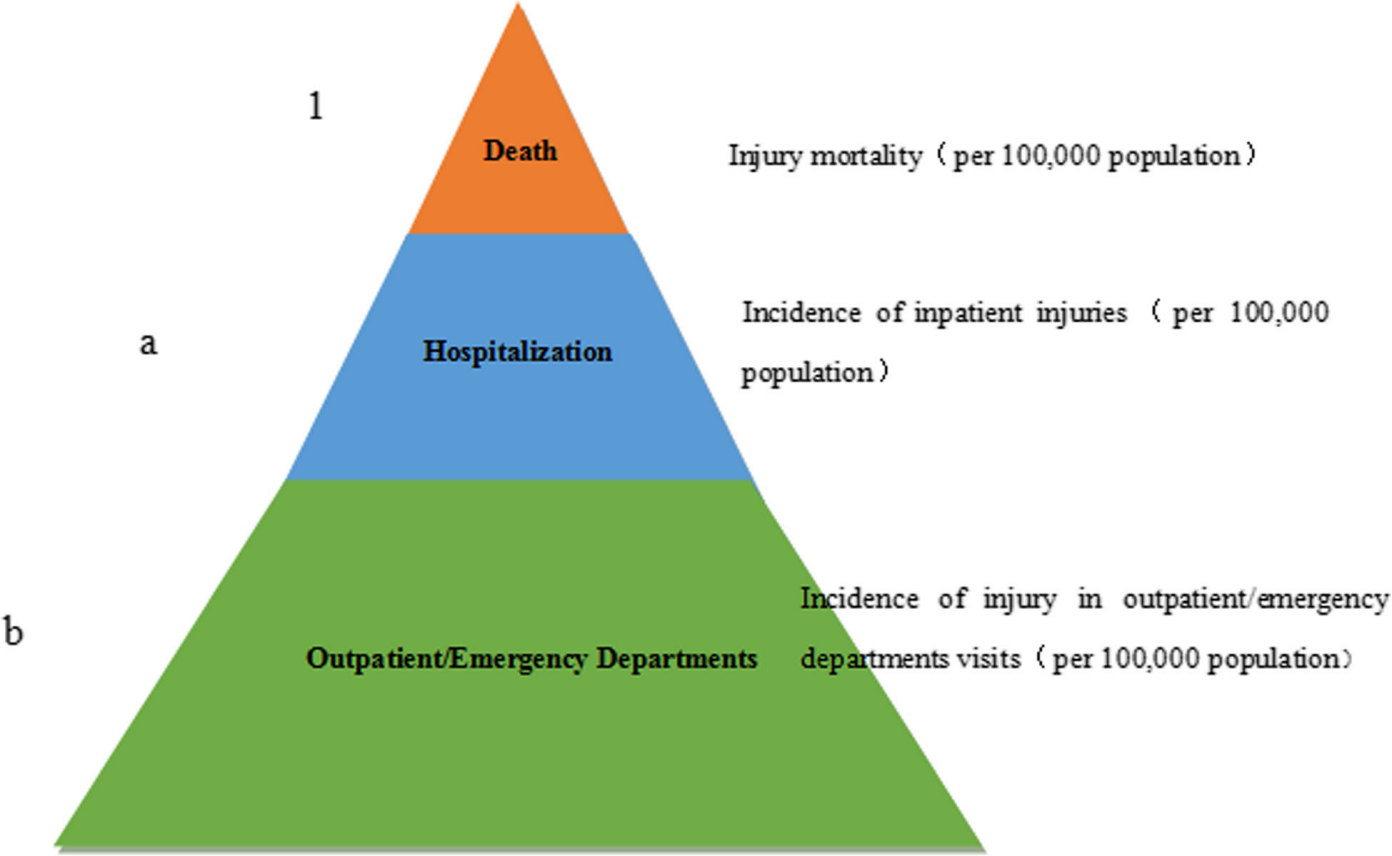
Schematic diagram of the injury pyramid ratios

The injury pyramid ratio (1, a: b) was calculated as shown in the figure: a = incidence of inpatient injuries/injury mortality, which means that every injury death occurred accompanying an injury hospitalization cases; b = incidence of injuries in outpatient/emergency department visits/injury mortality, which represented every injury death that occurred accompanying b outpatient/emergency department injury cases (Fig. 1).

### Statistical indicators

Injury mortality (per 100,000 population) = total deaths (due to injury) in a certain period / number of resident population × 100,000 .

Incidence of inpatient injuries (per 100,000 population) = number of inpatient injuries cases / number of resident population × 100,000. Because the current resident area was unknown for 1175 cases of hospitalized injuries, these cases were split according to the proportions of the average populations of urban and rural areas in 2013–2017 between the number of urban and rural hospitalizations for injuries to calculate the incidence of injury hospitalizations.

Incidence of outpatient/emergency department injury visits (per 100,000 population) = total number of outpatient emergency department injury visits (n / p) / number of resident population × 100,000.

*p*: Community survey results were used to estimate the probability that injury patients visit outpatient/emergency departments at NISS monitoring hospitals. Data from a retrospective survey based on the entire population conducted in the same city in 2011 [14] showed that of the 2060 people who were surveyed in the community, 350 reported an injury within 6 months of survey administration, and 88 of them visited outpatient/emergency departments at NISS monitoring hospitals, *p* = 0.251. *n*: Number of injury cases that saw a doctor at an outpatient/emergency department in an NISS monitoring hospital.

Statistical Package for the Social Sciences (19.0; IBM Corp Armonk, NY, USA; SPSS) was used for statistical analysis.

## Results

### Injury mechanisms

The top three mechanisms for injuries in outpatient/emergency departments for children and adolescents were falls (52.02%), animal bites (14.57%), and blunt injuries (10.60%). The top three injury mechanisms for hospitalizations were falls (37.33%), road traffic injuries (17.87%), and fire/burns (14.29%), while the top three mechanisms for injury deaths were drowning (32.91%), road traffic injuries (20.25%) and falls (13.92%). In addition, cuts, poisoning, asphyxiation, self-harm/suicide and other events also had a large impact on the health of children and adolescents (Table 3).

**Table 3:**
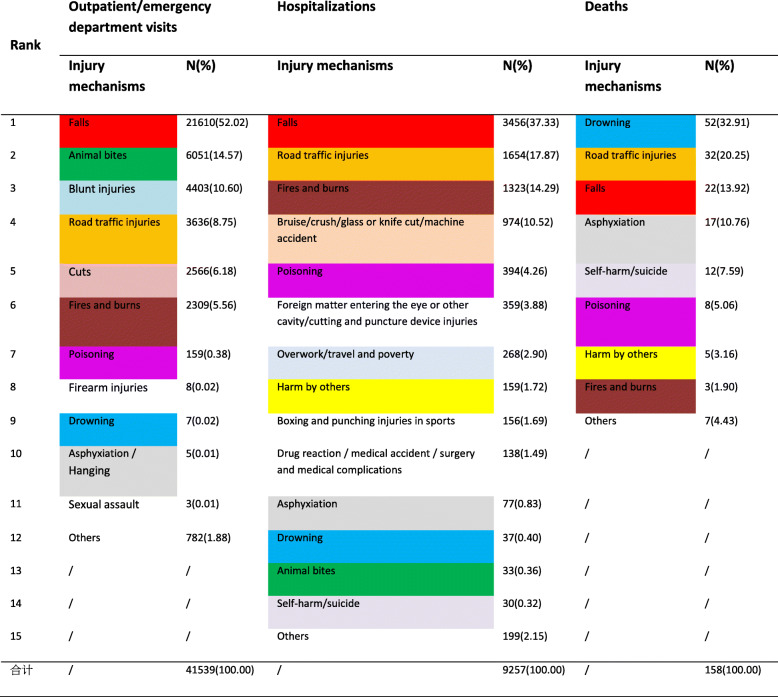
Injury mechanisms among children and adolescents in Zhuhai City, China

Falls were the leading mechanism of outpatient/emergency department visits and hospitalizations for injured children and adolescents in urban and rural areas. Animal bites to children and adolescents in urban areas were a secondary mechanism of outpatient/emergency visits, while in rural areas, the secondary mechanism was road traffic injuries. Among the mechanisms of injury deaths, road traffic injuries ranked first in urban areas, while drowning ranked first in rural areas.

Among the mechanisms of injuries in outpatient/emergency department visits for boys and girls, the top two were falls and animal bites; blunt injuries accounted for the third mechanism for boys, and road traffic injuries accounted for the third mechanism for girls. Boys and girls had the same top five ranking injury mechanisms among injury hospitalizations. Among the mechanisms of injury deaths, drowning and road traffic injuries accounted for the first two for boys and girls, and the third ranking mechanisms were falls for boys and asphyxiation for girls.

Comparing the injury spectra of children and adolescents in different age groups, it was found that in the outpatient/emergency department visits, in addition to falls, which were the first ranking mechanism of injury in each age group, fires and burns for children aged 0 ~ 2 years old, animal bites among those ages 3–14 years, and blunt injuries among those ages 15–17 years old were the secondary injury mechanisms. Among injury hospitalizations, the top three ranking injury mechanisms in all age groups were falls, road traffic injuries, fires and burns (in different order). Regarding the injury death mechanisms, drowning ranked first for children and adolescents aged 0 to 11 years, the second mechanism of death for children aged 0 to 2 years was suffocation, and the second mechanism of death for children aged 3 to 11 years old was road traffic injuries. The first mechanism of injury death among children and adolescents aged 12 to 17 years was road traffic injury, the second was self-harm/suicide, and the third was drowning (Table 4).

**Table 4:**
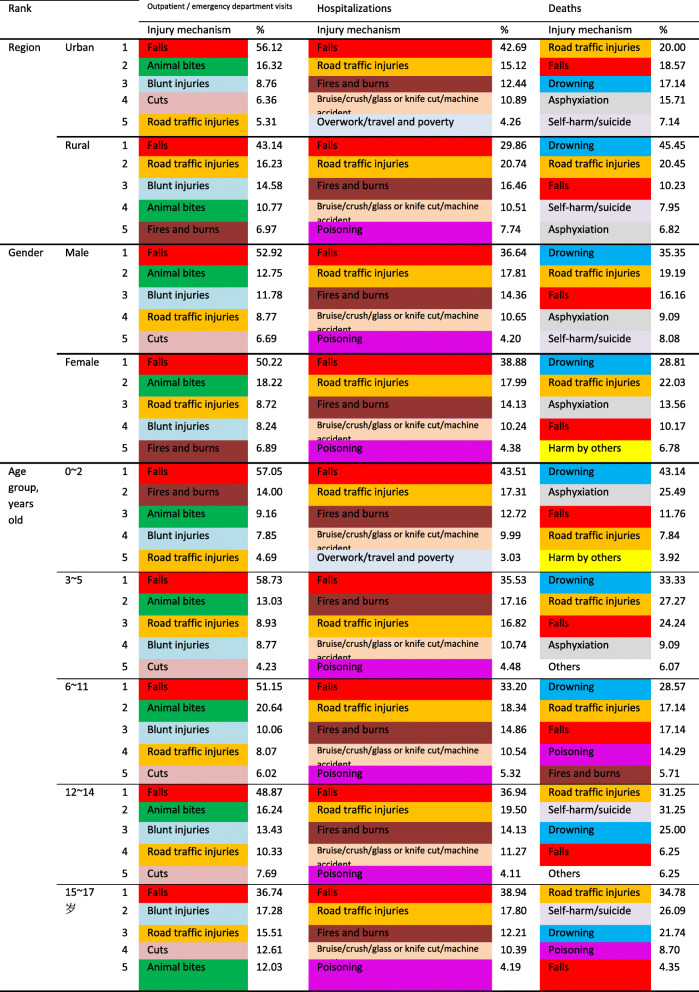
Injury mechanisms among different regions, genders, and age groups of children and adolescents in Zhuhai City, China

### Injury pyramid ratios

The incidence of outpatient/emergency department injury visits for children and adolescents was 11,210.87/100,000, the incidence rate of injury hospitalization was 627.09/100,000, and the injury death rate was 10.70/100,000. For each injury death, there were 59 hospitalized injuries and 1048 outpatient/emergency visits for injuries. The injury pyramid ratios varied among different regions, sex, and age groups of children and adolescents. The injury pyramid ratio for drowning was 1:0.7:0.5 (Each injury death that occurred was accompanied by 0.7 injury hospitalization and 0.5 outpatient/emergency injury visit) and in the shape of an inverted triangle, which was different from falls (1:157:3914), road traffic injuries (1:52:452) and self-harm/suicide (1:3:20) (Table 5).

**Table 5:** Injury pyramids ratios of children and adolescents in Zhuhai City, China

		Incidence of outpatient/emergency department injury visits (per 100,000 population)	Incidence of inpatient injuries (per 100,000 population)	Injury mortality (per 100,000 population)	Injury pyramid ratios
Total		11,210.87	627.09	10.70	1:59:1048
Region	Urban	13,688.03	633.32	8.46	1:75:1618
	Rural	8050.49	619.14	13.57	1:46:593
Gender	Male	13,638.37	790.77	12.22	1:65:1116
	Female	8259.64	428.08	8.86	1:48:932
Age group, years old	0 ~ 2	16,820.13	867.94	22.34	1:39:753
	3 ~ 5	19,051.17	899.61	16.43	1:55:1160
	6 ~ 11	11,257.02	622.70	8.06	1:77:1397
	12 ~ 14	7286.77	485.35	6.95	1:70:1048
	15 ~ 17	6055.17	430.51	6.02	1:72:1006
Injury mechanisms	Falls	5832.28	234.12	1.49	1:157:3914
	Road traffic injuries	981.31	111.77	2.17	1:52:452
	Self-harm/suicide	16.46	2.03	0.81	1:3:20
	Drowning	1.89	2.51	3.52	1:0.7:0.5^a^

^a^The injury pyramid for drowning was in the shape of an inverted triangle, with each injury death that occurred was accompanied by 0.7 injury hospitalization and 0.5 outpatient/emergency injury visit

## Discussion

The average injury mortality rate of children and adolescents in Zhuhai City from 2013 to 2017 was 10.70/100,000, which was lower than the injury mortality rate of global entire population (66.90/100,000) [15], higher than the injury mortality rate of children and adolescents in HICs (7.70/100,000), and lower than the injury mortality rate of children and adolescents in LMICs (37.20/100,000) [16], while the average injury mortality of children and adolescents in Zhuhai City was also lower than the total injury mortality of children aged 0–17 in China in 2014 (22.90/100,000) [4] and lower than the injury death rate of the entire population in Guangdong Province in 2015 (43.11/100,000) [17]. Drowning and road traffic injuries were the main causes of death among children and adolescents in Zhuhai City, which is consistent with the major causes of death among children in China [18]. Road traffic injuries are the leading cause of child injury deaths worldwide, followed by drowning [3]. The main causes of child injury deaths in the United States were road traffic injuries and firearm injuries. There are differences in injury mortality patterns in different countries and regions.

Drowning is one of the leading causes of death among children aged 5 to 14 years old in China [19]. The average drowning mortality of children and adolescents in Zhuhai was 3.52/100,000, which was lower than the national average mortality (7.46/100,000) in China [4]. This difference may be related to the effectiveness of Zhuhai City’s interventions to reduce child drowning in recent years, including health education for students, teachers, and guardians, the establishment of a school safety education leadership group, and the implementation of a responsibility system to sign responsibility letters, among other measures. Drowning in Malaysia was estimated at 5 per 100,000 people, including nondeath cases [20]. The overall drowning mortality rate in Nepal was 1.90 per 100,000, but 53.00% of drownings occurred in people under 20 years old, and most drownings occurred in rivers (natural bodies of water) [21]. Drowning ranked first in injury deaths among children and adolescents in rural areas in Zhuhai City. Drowning is closely related to temperature, environmental risks, and water-related risk activities [22], especially in rural areas of Zhuhai, where there are many rivers and ponds.

The average death rate attributable to road traffic injuries for children and adolescents in Zhuhai was 2.17/100,000, which was lower than the national death rate of road traffic injuries for children and adolescents (6.66/100,000) [4]. This difference may be related to Zhuhai City’s regulations prohibiting riding motorcycles and electric vehicles, which are available in only a few cities across the country, and the popularity of child safety seats in recent years. From 1991 to 2015, the death rate attributable to road traffic injuries among children and adolescents aged 1 to 14 years in Ireland decreased from 2.10/100,000 to 0.32/100,000 [23]. From 2006 to 2012, the incidence of road traffic injuries in Canadian children was 70.91/100,000, and the hospitalization rate for injuries decreased from 85.51/100,000 to 58.77/100,000 [24]. With the implementation of the global “traffic safety promotion”, the use of seat belts and appropriate child safety seats has steadily increased, and the production of safer cars, better road construction, and increased public awareness of driving risk behaviors have reduced road traffic injuries among children and adolescents [25, 26]. Although road traffic injuries for children and adolescents in Zhuhai City were lower than the mortality rate for children and adolescents in China, road traffic injuries were still the main cause of death, hospitalization and outpatient/emergency department visits, and there is still much more space for improvement.

The global burden of disease estimates indicate that 172 million falls result in short- or long-term disability annually [27]. A cohort study in Brazil found that falls were the most commonly reported injuries in all age groups among children [28]. Falls among children and adolescents in Zhuhai City were the main reason for hospitalization and outpatient/emergency department visits. A large household survey covering more than one million people in Bangladesh in 2013 found that nonfatal falls were the most common injuries [29].

Animal bites were one of the main reasons for children and adolescents to visit the outpatient/emergency department for injuries. A study showed that the incidence of animal bites in China was 245.05 per 100,000 in 2016 [30]. Although animal bites are mostly minor injuries, as increasing numbers of pets enter the family, the problems caused by animal bites cannot not be ignored.

A multicountry survey of children aged 14–17 years found that 8.90% of girls and 2.60% of boys reported self-harm incidents in the past year [31]. Studies by Muehlenkamp [32] and Swannell [33] showed that self-harm/suicide behavior was prevalent in adolescents in clinical and community samples. The suicide mortality rate of children and adolescents in Zhuhai City was 0.81/100,000. Most of them were older children aged ≥12 years, and the suicidal tendency increased with age. The self-harm/suicide rates of children and adolescents are rising in many high-income countries [34–36]. The development trajectory, cultural background, risk factors and mechanisms, and independent diagnosis of self-harm/suicide among children and adolescents in China will be the focus of future research.

Studies in several countries have shown that rural children and adolescents have higher injury mortality rates than urban children [37, 38]. Rural children and adolescents in Zhuhai had an injury mortality rate of 13.57 per 100,000, which was 1.60 times that of urban areas. Road traffic injuries were the leading cause of injury deaths among children and adolescents in urban areas, and drowning injuries were the leading cause of injury deaths among children and in rural areas. These urban-rural differences could be caused by environmental factors, residents’ behavioral factors, different infrastructure investments, and differences in access to emergency medical services [39].

The mechanisms of injury deaths, injury hospitalizations, and outpatient/emergency department injury visits showed obvious age characteristics, and there were differences in the injury spectra at different stages of child development. A study in the United States showed that drowning was the most common cause of death for children aged 1 to 4 years, and the causes of death from injuries among those aged 10 to 19 years was mainly road traffic injuries, firearm injuries and suffocation [40]. The main mechanisms of injury hospitalizations for children aged 0–16 years in Australia were violence, poisoning, burns and road traffic injuries [41]. Falls were the most common mechanisms of nonfatal injuries to children in Nepal, followed by burns in preschool children, and road traffic injuries were the most likely mechanisms of injury during adolescence [42]. For outpatient/emergency department injury visits and injury hospitalizations, falls were the leading cause of injury for all age groups in Zhuhai City. Children in the younger age group had more outpatient/emergency department visits because of burns and animal bites. In the older age group, there were more blunt injuries and cuts. Drowning was the main cause of injury death among young children, and road traffic injuries were the main cause of injury death among children and adolescents in older age groups.

As a model for injury research, the injury pyramid can visually show the relative effects of fatal and nonfatal injuries [43]. This study showed that, for every injury death among children and adolescents in Zhuhai, there were 59 injury hospitalizations and 1048 outpatient/emergency department visits for injuries. Similar to the injury pyramid (1:36:1014) described by Lee [9] for children and adolescents ≤19 years old living in Massachusetts, USA, Ballesteros [44] also found that every injury death among children in the United States was accompanied by approximately 1000 injury-related outpatient/emergency department visits. Compared with the injury pyramid for South Korean adolescents (1:162:492) [10], for every injury death, the percentage of injury hospitalizations of children and adolescents in Zhuhai City was small, while the proportion of outpatient/emergency department visits was high. This difference may be due to different medical guarantees in different countries, and China may have a relatively heavy burden of hospitalization costs, which limits hospitalizations. In addition, the severity of the injury and the timeliness of treatment can also affect hospitalizations.

Compared with the whole population injury pyramid, the rate of outpatient/emergency department injury visits among children and adolescents was greater. The injury pyramid ratio for unintentional injuries among the entire South Korean population was 1:54:231 [8]. The injury pyramid ratio in the entire population in Missouri and Nebraska was 1:10:147 [11]. The injury pyramid ratio in Iran, based on a household survey, was 1:50:646 (deaths:hospitalizations:all injuries) [12]. Studies in the same country still showed higher rates of injury among children and adolescents seen in the outpatient/emergency departments. For example, the injury pyramid ratio for children who were ≤ 10 years old and came from Alberta, Canada, was 1:73:1612 [45], and the injury pyramid ratio for children who were ≥ 12 years old and came from Ontario, Canada, was 1:25:363 [46]. Fatal injuries were at the top of the injury pyramid, but minor nonfatal injuries were more common in children and adolescents than adults, and the burden of minor nonfatal injuries cannot be ignored.

This study showed that rural children and adolescents had higher injury mortality rates than urban children and adolescents, while the incidence of outpatient/emergency department visits for injuries was higher in urban areas than in rural areas. Compared to the urban injury pyramid (1:75:1618), the rural injury pyramid has a narrower shape (1:46:593), which was related to the difference between injury-related death rates, hospitalization rates, and injury mechanisms in urban and rural areas. Incidence/mortality was higher at all levels in boys than in girls. The frequency of fatal and nonfatal injuries was higher in males than in females. Research in South Korea found that the injury pyramids of children and adolescents aged 0 to 6 years (1:151:3657), 7 to 12 years (1:280:3011), and 13 to 18 years (1:132:594) showed that the younger the age, the wider the bottom of the pyramid [8]. This study showed that the bottom of the injury pyramid was the widest among participants aged 6 to 11 years; with each injury death that occurred, there were the most injury hospitalizations and outpatient/emergency department injury visits. The highest injury mortality was observed in children aged 0 to 2 years, and this injury pyramid had the narrowest bottom (1:39:753). The rest of the children aged 3 to 5 years (1:55:1160), 12 to 14 years (1:70:1048), and 15 to 17 years (1:72:1006) had similar pyramid shapes. Injuries were more lethal to children in younger age groups.

Wadman MC [11] believed that according to the lethality of the injury mechanism, injury data will lead to 3 different pyramid types, with an inverted pyramid indicating high-lethality injuries (C), a rectangular pyramid indicating mid-level-lethality injuries (B), and a typical pyramid indicating low-lethality injuries (A). The ratio of injury pyramid of self-harm/suicide among South Korean youth was 1:7:15 [10], showing a steep pyramid (type C); this was similar to the injury pyramid of self-harm/suicide among children and adolescents in Zhuhai City (1:3:20), indicating that self-harm/suicide had considerable lethality. The drowning injury pyramid was in the shape of an inverted triangle, indicating that, for every injury death that occurred was accompanied by 0.7 injury hospitalization and 0.5 outpatient/emergency injury visit. It was observed that self-harm/suicide and drowning had very high mortality rates, but the numbers of outpatient/emergency department visits for these mechanisms were very small, resulting in steep and narrow pyramids.

The mortality rate of road traffic injuries among South Korean youth was 3.00/100,000, with an injury pyramid ratio of 1:195:341 [10]. The death rate of road traffic injuries for children and adolescents in Zhuhai was 2.17/100,000, and the injury pyramid ratio was 1:52:452. The shape of the injury pyramid for road traffic injuries was type B, indicating that the mortality rate was low, but the degree of injury was high. Type B injury pyramids have a cumulative effect on the use and cost of the medical system that is far greater than type C. The injury pyramid ratio for falls among children and adolescents in Zhuhai was 1:157:3914. Falls had a low mortality rate, but the outpatient/emergency department visits were large in scale, forming a type A pyramid. An injury mechanism with high lethality can reduce the occurrence of the injury itself through primary prevention; however, although the fatality rate of falls was very low, the scale of occurrence was large, and the consumption of medical resources may be more serious.

### Limitations

The injury hospitalization data used in this study were derived from a medical information management platform. We did not obtain the nature of injury codes, but only the external cause codes from hospital data systems, and the external cause codes, which we mainly analyze, may be misclassifications. However, due to a problem with platform integration, only 6 medical institutions were included in 2013; this number increased year by year until it reached 24 included institutions in 2017 (essentially covering all medical institutions with the ability to treat injuries). Therefore, the incidence of injury hospitalizations may be underestimated. In addition, there was a lack of information on some subjects’ current addresses, and the urban or rural area residence of some subjects could not be determined. The incidence of outpatient/emergency department injury visits was estimated based on the results of community surveys in the literature; these estimation methods may make the estimation efficiency low.

Most minor injuries were difficult to estimate because they did not seek health care services. In addition, there may be injury sequelae among survivors of severe injuries, leading to even more important estimates of disability levels. Ideally, the injury pyramid should include untreated cases and injuries caused by disability, which is also a direction for future efforts.

## Conclusions

The injury mechanisms were different among outpatient/emergency department visits, hospitalizations, and deaths among children and adolescents. Falls, animal bites, road traffic injuries, fires and burns were the main injuries for which children and adolescents sought medical treatment; drowning and road traffic injuries were the main causes of injury deaths. The sexes have different injury mechanisms at different stages of child development, and interventions should be formulated accordingly.

For every case of injury death among children and adolescents, there were nearly 60 hospitalized injuries and thousands of injury-related outpatient/emergency department visits. Minor nonfatal injuries were more common in children and adolescents. The size and shape of the injury pyramid varied by age, sex, and region; that is, there were differences in the severity and extent of the injuries. The injury pyramids suggested that self-harm/suicide and drowning had very high mortality rates. Road traffic injuries had a low mortality rate and a high degree of injury; falls had a low mortality rate, but the scale of outpatient/emergency visits was large.

## Data Availability

The datasets used and/or analysed during the current study are available from the corresponding author on reasonable request.

## Abbreviations

HICs: High-income countries
LMICs: Lower-middle- and Upper-middle-income countries
NISS: National Injury Surveillance System
